# A Hierarchical Etched Grid for Correlative Chemical Imaging

**DOI:** 10.1021/cbmi.5c00231

**Published:** 2026-03-04

**Authors:** Natalie S. W. Arigundiya, Jake Brooks, Peter Sykes, Seojin Lee, Naomi P. Visanji, Christopher M. Morris, Kalotina Geraki, Joanna F. Collingwood

**Affiliations:** † Analytical Science Centre for Doctoral Training, 2707 University of Warwick, Coventry CV4 7AL, United Kingdom; ‡ School of Engineering, 2707 University of Warwick, Coventry CV4 7AL, United Kingdom; § 120796 Diamond Light Source, Harwell Science and Innovation Campus, Didcot OX11 0DE, United Kingdom; ∥ Rutherford Appleton Laboratory, 275294 Scitech Precision Limited, Harwell Science and Innovation Campus, Didcot OX11 0QX, United Kingdom; ⊥ Tanz Centre for Research in Neurodegenerative Diseases, 7938 University of Toronto, Toronto, Ontario M5T 0S8, Canada; # Department of Laboratory Medicine and Pathobiology, 7938 University of Toronto, Toronto, ON M5T 0S8, Canada; ∇ Krembil Brain Institute, University Health Network, Toronto, ON M5T 0S8, Canada; ○ Edmond J. Safra Program in Parkinson’s Disease and the Morton and Gloria Shulman Movement Disorders Clinic, Rossy Progressive Supranuclear Palsy Centre, Toronto Western Hospital, Toronto, ON M5T 2S8, Canada; ◆ Newcastle Brain Tissue Resource, Institute of Neuroscience, 5994 Newcastle University, Newcastle-upon-Tyne NE4 5PL, United Kingdom

**Keywords:** correlative microscopy, synchrotron X-ray fluorescence, metallomics, femtosecond laser etching, trace metal analysis, nondestructive, biomedical imaging

## Abstract

Correlative microscopy linking synchrotron X-ray fluorescence (SXRF) with optical imaging is valuable for contextualizing chemical element distributions in biology. The spatial correlation necessary to achieve this presents fundamental challenges and can be a significant constraint on accuracy and data interpretation. We present a technical solution based on a finder grid concept, optimized for SXRF correlative studies of metals in biological tissues, with scope for wider adaptation and application. A hierarchically patterned fiducial system was directly etched onto spectroscopically clean quartz substrates via femtosecond laser ablation. This design enables improved correlation among SXRF, optical imaging, and histological staining over a greater range of length scales than conventional registration methods such as the use of tissue architecture from serial sections and the use of electron-microscopy-resolution finder grids and applied fiduciary markers that can introduce XRF-signal-dominating levels of elements such as copper, nickel, gold, and titanium. We present two quartz finder grid formats: a microgrid and a nanogrid design. We demonstrate their utility for rapid ROI relocalization and same-section correlative workflows using human brain tissue. The etched quartz finder grid approach facilitates rapid and reproducible ROI relocalization and alignment across instruments, particularly where integral fiducial markers are sparse or ambiguous.

## Graphical abstract

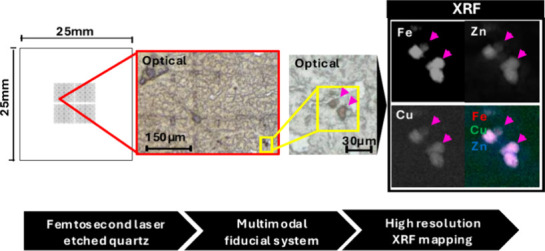

## 1 Introduction

Spatially resolved metallomics is providing unparalleled insight into the role of metals in biological systems, including challenging areas such as neurological health and disease. Synchrotron X-ray fluorescence (SXRF) microscopy is a cornerstone technique for the nondestructive multielemental mapping of biological tissues.^1^ Interpretation of the resulting elemental maps is typically informed by correlation with staining of the same or adjacent tissue sections that have been histologically stained, providing crucial morphological and cellular context.^2^

A robust correlative approach benefits from fiducials that can be readily visualized in the imaging plane across modalities. In practice, however, implementing a shared fiducial system that is visible in multiple reflection- and fluorescence-based imaging modalities is challenging due to conflicting geometries, materials, thicknesses, and contrast requirements. Synchrotron beamline endstations also differ substantially in mounting geometry, allowable substrate thickness, working distance, and field of view.^3^

Here, we propose a transferable design framework based on hierarchical coordinate logic. Within this framework, the approach is optimized for hard X-ray fluorescence (XRF) imaging operating in reflection fluorescence geometries, which are now widely available at major synchrotron facilities, typically spanning micro- to nanolength scales.^4^ Micro-SXRF beamlines commonly provide micrometer-scale spatial resolution, with motorized stages enabling millimeter-scale mapping. In contrast, nano-XRF beamlines achieve spatial resolutions down to tens of nanometers but operate over more restricted mapping areas, placing greater emphasis on precise navigation and coordinate-based targeting. Accordingly, the grid coordinate system presented in this Technical Note does not aim to address all possible modalities; rather, it is intentionally optimized for reflection fluorescence workflows where precise in-plane registration and mechanical robustness are critical. The design is demonstrated at two beamlines equipped, respectively, for micro- and nano-XRF imaging: Diamond Light Source I18 and I14.

### 1.1 Current Correlative Imaging Approaches

For decades, there has been reliance on using tissue landscape or commercially available finder grids and other fiduciary systems made from metals such as nickel (Ni), copper (Cu), gold (Au), and titanium (Ti).^5,6^ This practice introduces a critical, yet often under-addressed, analytical compromise: the introduction of strong exogenous signal from elements that are themselves targets of SXRF. In these instances, such signal elevates background, obscures genuine biological signals, and can introduce spectral artifacts including sum peaks, diffraction peaks, and escape peaks, causing smaller peaks to be overestimated or missed entirely.^7,8^ This issue is particularly acute in microprobe SXRF, where it is often impractical to avoid intersecting the beam with metallic fiducials, rendering certain elements effectively inaccessible despite being within the measurable energy range.

Additionally, the length scales afforded by conventional substrates used for nanoscale SXRF imaging can impose fundamental limitations on correlative workflows in this application area. The smaller-format substrates (mounting area typically <3 mm in diameter) typically require the sample to be dissected within anatomical regions of interest. This introduces a number of constraints, and in particular, the relationship with surrounding tissue, adjacent structures, or models of disease is very difficult to preserve accurately in this context.^9^ However, for larger-format samples retaining anatomical areas, researchers lack robust fiducial markers that are not a potential vehicle for contamination, often relying on imperfect natural tissue landmarks such as blood vessels or section damage (such as a scratch or hole) for navigation, a process that can be subjective, error-prone, and challenging when working at high magnification differences and across a variety of imaging instruments.^10^ In well-preserved tissue, distinctive intrinsic tissue landmarks may be absent altogether, complicating ROI relocalization and image registration. Previous studies have addressed this limitation by deliberately introducing artificial features, such as pinholes, into the tissue sections to serve as fiducial markers.^11^ A further challenge is that researchers using focused beam SXRF to analyze tissue sections may default to evaluating adjacent paired tissue sections on different substrates (one for SXRF, and the adjacent one histologically stained), with scope to introduce additional registration uncertainty.^12^

Here, we introduce a practical solution to address a number of these challenges through a nondestructive, reproducible fiducial marker system engineered to enable robust localization and registration without intentional tissue damage while minimizing the introduction of signal from elements relevant to biological tissue. By employing femtosecond laser ablation to etch hierarchical grid patterns directly into well-defined, spectroscopically clean, rigid quartz substrates, we create durable, high-contrast fiducials without introducing any additional chemical signal. We outline a correlative workflow in which etched quartz grids enable rapid, accurate, and reproducible ROI relocalization and coordinate-based registration across optical microscopy and SXRF imaging, and we demonstrate same-section correlation with histological staining using micro- and nano-SXRF examples.

## 2 Materials and Methods

### 2.1 Etched Grid Design Fabrication

All manufacturing of the grids presented in this Technical Note was performed by Scitech Precision Ltd. using a femtosecond laser ablation system (laser machining was performed at a 50 kHz repetition rate; 6.4 W at the laser corresponded to ∼800 mW delivered at the workpiece for the standard-thickness quartz microscope slides, while 0.64 W at the laser corresponded to ∼80 mW at the workpiece for the thinner quartz coverslips). The parameters were optimized to produce continuous lines with a maximum thickness of 13–15 μm, without subsurface damage to the quartz, as illustrated in Figure 1.

**Figure 1:**
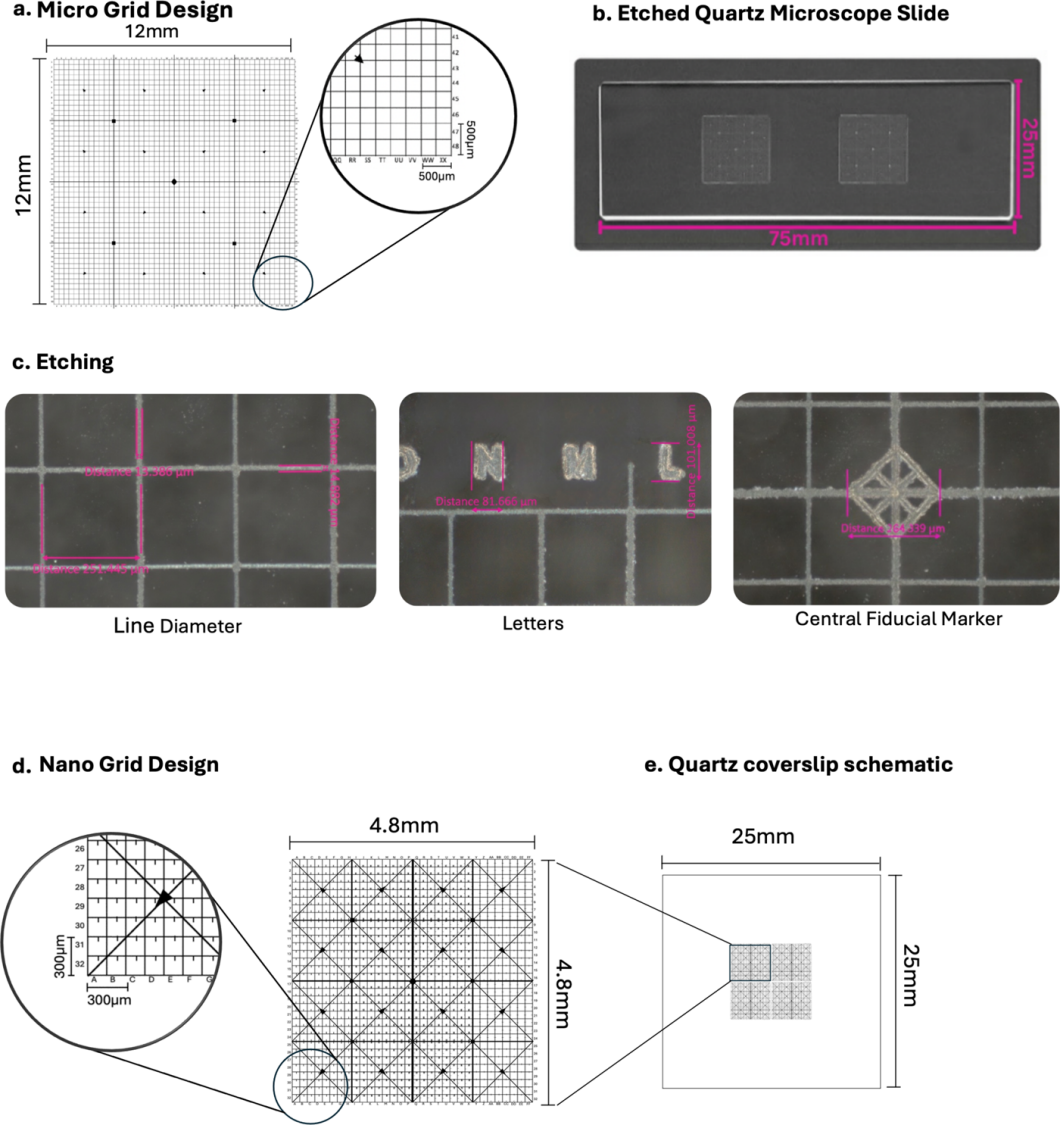
Hierarchical grid designs for SXRF. (a–c) Micro Grid Design engineered on standard-sized fused quartz slides (25 × 75 × 1 mm^3^) for microfocus XRF. The slide carries a 12 × 12 mm^2^ array of 250 × 250 μm^2^ squares with an alphanumeric coordinate system for macroscopic sample tracking; each square includes minor fiducial markers for localized registration. Etching dimensions are specified for grid lines, alphanumeric labels, and the major fiducial marker. (d, e) Nano Grid Design fabricated on quartz coverslips (25 × 25 × 0.15 mm^3^) for data collection. The smaller format of the quartz coverslip facilitates mounting at the Hard X-ray nanoprobe I14 beamline at Diamond Light Source, but in practice the same grid patterns could be etched onto a standard-sized slide. Each coverslip contains four identical 4.8 × 4.8 mm^2^ master grids; each master grid is subdivided into 32 labeled 150 × 150 μm^2^ squares, which are further partitioned into 16 subsquares with directional arrows, enabling precise navigation and reliable relocation at submicron resolution.

Two grid systems, to accommodate micro- and nanoscale imaging, were designed and etched onto the chosen quartz substrates (Figure 1). In the examples shown, multiple copies of the micro- and nanoscale designs were etched onto each substrate, but the area covered or combination of grids on a single substrate could be adapted according to study design requirements. Here, the two substrate formats were developed to balance (i) correlative workflow requirements for large tissue sections and routine optical handling, and (ii) the mechanical and field-of-view constraints of beamlines (I18 and I14). For microprobe work, standard microscope-slide dimensions provided robust handling, compatibility with common histology workflows, and support for large-format sections; the grid was etched only where needed for navigation and alignment, reducing fabrication time and cost. For nanoprobe work, the sample environment typically imposes stricter constraints, offering a restricted mapping field of view; the denser hierarchical grid format therefore prioritizes unambiguous, high-precision relocation of small regions of interest (Figure 1e). The nanogrid design was etched into coverslips. Although the nanogrid design could be etched onto a standard-sized quartz slide, it was applied to quartz coverslips in this study to facilitate downstream handling by other modalities with sample stage constraints. However, thinner substrates offer additional movement in tight working lengths, where there is an increased collision risk with beamline optics. Importantly, the nanoformat grid design is transferable to microprobe beamlines, whereas the microgrid format is less suitable for nanoprobes because the wider fiducial spacing (250 μm) provides a length scale on the microgrid format, which is a constraint to navigation at the nanoscale, so it is better suited to correlative imaging with other microscale techniques such as optical microscopy over large sample areas.

The example design for the I14 nanoprobe at Diamond took into account the available field of view and dimensional requirements for sample mounting. The I14 on-axis field of view is approximately 3 mm × 3 mm^2^, which covers only a portion of a single master grid. Human brain tissue sections were typically derived from tissue blocks of approximately 1 × 1 cm^2^. The designs presented here optimized slide area use for this sample format and were found to be particularly effective when mounting sets of adjacent sections, thereby maximizing sight of the sections under the constraints of stage travel and the alignment camera field of view at the beamlines used. This is particularly helpful for facilitating mapping of multiple sections without slide changes, as well as reducing repeated repositioning, and helped minimize excessive counting from large-area nanoscale survey mapping (which becomes impractically time-consuming at very small step sizes of a few hundred nanometers). The design criteria here were: (i) beamline mounting/on-axis viewing constraints, (ii) compatibility with histology handling and optical imaging, (iii) rapid and reproducible ROI localization using hierarchical coordinates, (iv) minimization of unnecessary large-area nanoscale survey mapping, and (v) manufacturability/cost-efficiency of etching while preserving a clear mounting area. Although 1 mm quartz slides are thicker than substrates traditionally preferred for SXRF (where thin Ultralene films are often chosen), the use of rigid quartz enabled a robust, durable registration system compatible with routine handling and histology, an approach that is difficult to implement reliably using fragile thin films or membranes. Furthermore, while thicker substrates can increase elastic/inelastic scatter and overall count rate,^13^ modern silicon drift detector systems with high-throughput digital pulse processing can operate stably at high count rates without appreciable loss of spectra resolution when appropriate dead-time monitoring corrections are applied.^14,15^

After quartz etching, a rigorous postetch cleaning protocol, adapted from Finnegan,^2^ was employed. Lint-free tissue was used to remove any obvious particulates and organic residues, and then the quartz slides were washed in 0.1% HCl in ethanol, thoroughly rinsed with ultrapure water (18.2 MΩ·cm), and dried at ambient temperature in a dust-free environment.

## 3 Results

### 3.1 Design and Production of Hierarchical Quartz Grid

The choice of substrate was critical to achieving a contamination-free baseline. We selected high-purity fused quartz over conventional soda-lime or borosilicate glass as optical-grade quartz is produced through manufacturing routes that yield an intrinsically pure material. Pre-etched quartz substrates were obtained from a commercial optics manufacturer (UQG Optics, Cambridge). The impurity levels are well-defined for this material, as shown in Table 1, and are below the ppm level for all elements except aluminium (Al) and titanium (Ti).^16^ In contrast, standard microscope glass slides made from soda-lime glass or borosilicate glass contain a variety of elements present at concentrations that can dominate signals from biological tissue sections, including silicon (Si), calcium (Ca), magnesium (Mg), aluminium (Al), and iron (Fe) in their oxide forms.^17^ The challenge with unwanted and heterogeneous signal contributions arising from glass slides is well-recognized throughout the synchrotron XRF community, and synthetic quartz is recommended by beamline scientists (such as at the Australian Synchrotron XRM beamline^18^) as an alternative where a rigid substrate that is transparent to optical wavelengths is beneficial.

**Table 1:** Evaluation of Measurement Background^a^

fused quartz chemical impurities typical mean value (ppm)												
Al	Co	Ca	B	Cu	Fe	K	Li	Mg	Mn	Na	Ni	Ti
18	0.01	0.4	0.03	0.02	0.2	0.6	0.4	0.02	0.06	0.8	0.02	1.2

a Supplier-reported bulk chemical impurities (ppm) for the synthetic fused-quartz; values are reported by the manufacturer (UQG optics) as typical concentrations.

To characterize contributions from the etched quartz substrate and the ambient/beamline environment, spectra were extracted from “off-tissue” regions of sections mounted on quartz slides patterned with the microgrid. Tissue-mounted slides were covered with an Ultralene layer as a protective barrier to minimize contamination from external sources during handling and measurement. SXRF measurements were performed at beamline I18 (11 keV incident energy, 40 μm step size, 0.5 s dwell time per pixel, under a helium atmosphere). Accordingly, the off-tissue spectra (quartz and Ultralene) capture the intrinsic quartz signal together with any extrinsic signal reaching the detector, such as scatter and secondary fluorescence arising from surrounding beamline components and residual air within the experimental enclosure. For each data set, spectra were taken from three representative locations across the map using 20 × 20 pixel regions of interest, both on-tissue and off-tissue, to assess spatial consistency and enable direct comparison of substrate and environmental contributions (Figure 2). The off-tissue spectra were dominated by the Si signal from the quartz substrate, with additional peaks attributable to Ar from residual air in the helium enclosure and a minor Fe contribution compatible with what we considered to be background/beamline scatter. In accordance with the supplier specifications for optical-grade fused quartz, no transition-metal peaks of biological relevance were detected above the beamline detection limit under the mapping conditions used here.^16^

**Figure 2:**
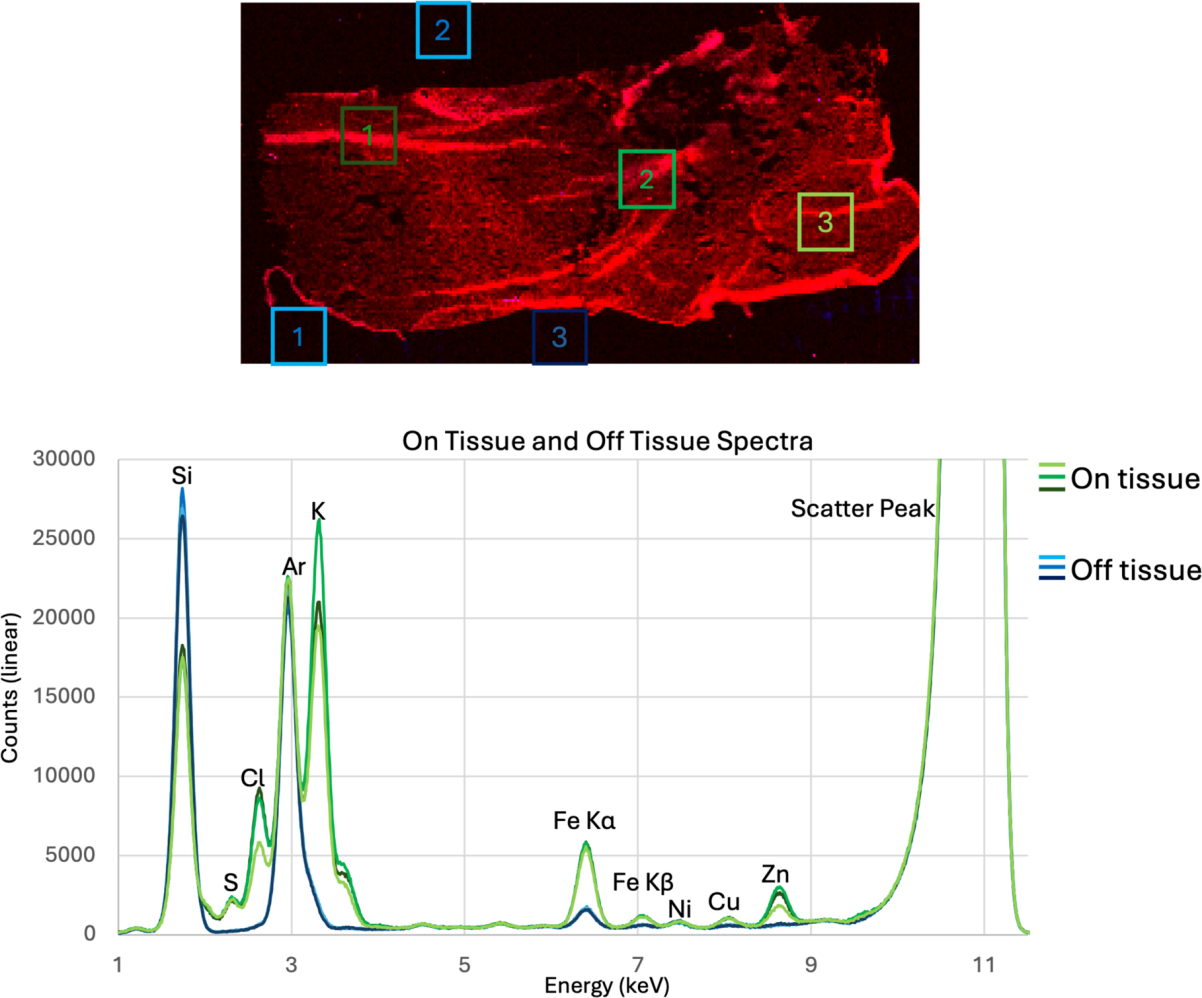
Tissue spectra and off tissue spectra. Representative SXRF spectrum acquired at beamline I18 (Diamond Light Source) from tissue sectioned onto etched quartz slides (11 keV incident energy, 40 μm step size, 0.5 s dwell time per pixel, under a helium atmosphere). Fused-quartz background (blue) and tissue (green) spectra from 3 areas of 20 × 20 px^2^ were obtained from PyMCA. The background spectrum is dominated by silicon (Si) from the substrate and argon (Ar). A small iron (Fe) peak is consistently observed in blank measurements and was confirmed by the beamline scientist to be primarily from the endstation background. Any constant background contribution can be readily subtracted. Blank spectra were acquired using the same acquisition parameters as the corresponding tissue maps to support background subtraction. Background peaks from other transition metals are at or below the beamline detection limit under these conditions.

To further confirm quartz contributions and to determine whether the Fe signal observed in off-tissue measurements (Figure 2) originated from the substrate or part of the beamline background, additional background spectra were acquired at beamline I18 using both quartz and Ultralene film (a polymer substrate frequently used in XRF) under identical acquisition conditions (11 keV, 2 μm step size, 0.5 s dwell, under a helium atmosphere) (Figure 3). Spectra acquired from fused quartz and Ultralene both exhibited Fe fluorescence. The observation of Fe in both materials, despite their very different compositions (inorganic SiO_2_ versus a thin polymer film), is most consistent with the constant background at the synchrotron endstation.

**Figure 3:**
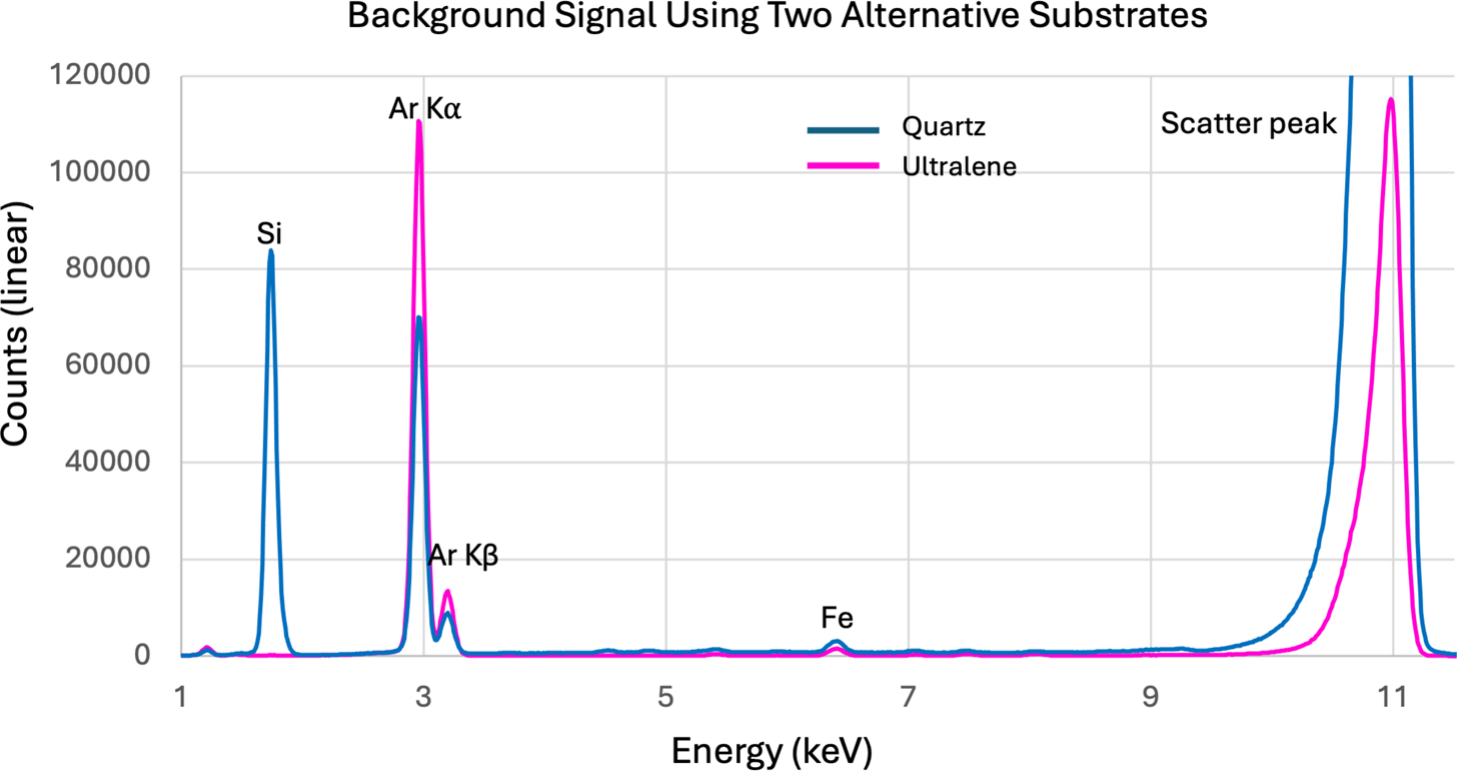
Background signal using two alternative substrates. Background XRF spectra acquired at beamline I18 from quartz and Ultralene film under matched conditions (11 keV, 2 μm step size, 0.5 s dwell time per pixel, under a helium atmosphere) and normalized to the primary scatter peak. The prominent Si peak in the quartz spectrum arises from the quartz substrate itself. The Fe Kα background is comparable for both substrates; the presence of a similar Fe Kα peak in Ultralene (polyethylene), which contains no iron, indicates the background signal at the beamline endstation rather than substrate-derived Fe. A small additional Fe contribution from the quartz substrate is observed as a slightly higher peak and is quantified in Table 1. For selected elements (e.g., Ar), the quartz substrate attenuates a portion of the background signal relative to Ultralene.

### 3.2 Biological Sample Preparation and ROI Relocalization

Unfixed, cryopreserved human brain tissue was sectioned (at 10–30 μm) to trial the fiduciary system and thaw-mounted onto the areas of the quartz slides where the etched grids were positioned, adhering without the need for any adhesive coating that could introduce an additional source of XRF signal. The subsequent correlative workflow is shown in Figure 4.

**Figure 4:**
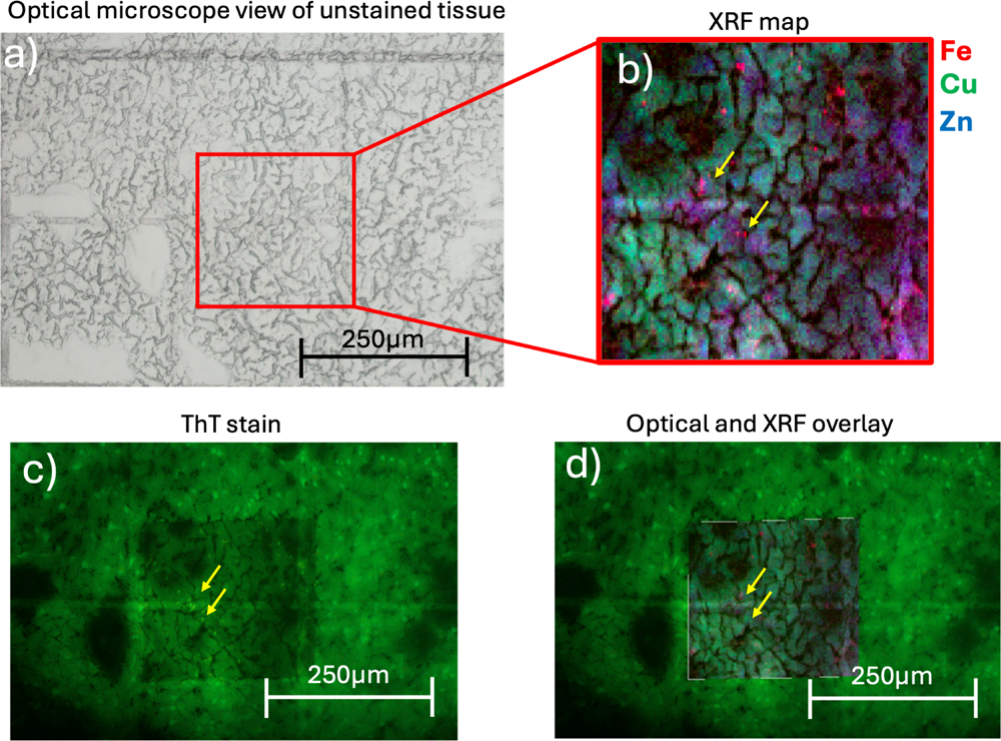
Post-SXRF correlative workflow using optical microscopy, SXRF mapping, and thioflavin-T staining. Schematic and representative images illustrating the postsynchrotron correlative workflow. (a) Tissue sections mounted on the etched quartz grid were first examined by optical microscopy to identify candidate regions of interest (ROIs) using native morphology and grid-referenced coordinates. (b) SXRF (250 x 250 μm, 2 μm step, and 0.5 s dwell) mapping was then performed to acquire elemental distributions from the selected ROIs. (c) Following SXRF analysis, the same tissue section was stained with thioflavin-T (ThT) to provide postmapping optical context. Post-SXRF staining is feasible; uptake of stains can be affected by beam-induced tissue damage with postirradiation tissue. (d) SXRF composite map shown in (b) overlaid onto ThT-stained image shown in (c). This workflow enables same-section correlation between elemental distributions and pathological features while avoiding the introduction of exogenous elemental background prior to SXRF however has its limitations.

Regions of interest (ROIs) were first selected on optical overview images acquired with the etched grid in the same focal plane as the tissue. When pre-SXRF staining was not performed, candidate ROIs were identified based on native tissue morphology, with input from experienced histologists identifying brain region boundaries. ROI positions were recorded using the alphanumeric grid coordinates and by noting the relationship between the ROI and the major and minor geometric fiducial markers. At the beamlines, the optically visible grid was positioned in the beam using the on-axis microscope camera. A coarse-resolution SXRF map was acquired over a large area encompassing the ROI to verify that the intended tissue features were in beam view and to refine the ROI position. A higher spatial resolution map was then acquired in the confirmed ROI. A higher-quality map was then followed by acquiring a map over the confirmed subregion at high resolution. The methods used for post-SXRF imaging followed the approach previously documented by our team for fragile, chemically unfixed tissues on quartz (e.g., Finnegan 2013).

This stage approach is particularly applicable on microprobe beamlines, where the larger field of view and faster survey mapping make it practical to relocalize ROIs without extensive prestaining. In contrast, on nanoprobe end stations, the effective field of view and high-resolution mapping at very small step sizes (e.g., 100 nm I14 (Diamond Light Source)) over large areas become prohibitively time-consuming. To address this problem in our nanogrid design, we employed a modified hyperphosphorylated tau immunostaining protocol to visualize tau pathology premapping which we were then able to relocate using tightly recorded grid-referenced coordinates on our grids at the nanoscale (Figure 5). Notably, SXRF workflows have often avoided prestaining because antibodies, buffers, and detection reagents can introduce exogenous metals that elevate background and confound interpretation of endogenous elemental signals. Here, we used a modified hyperphosphorylated tau immunostaining protocol specifically designed to minimize metal contamination. Details of the modified protocol are the subject of a forthcoming publication.

**Figure 5:**
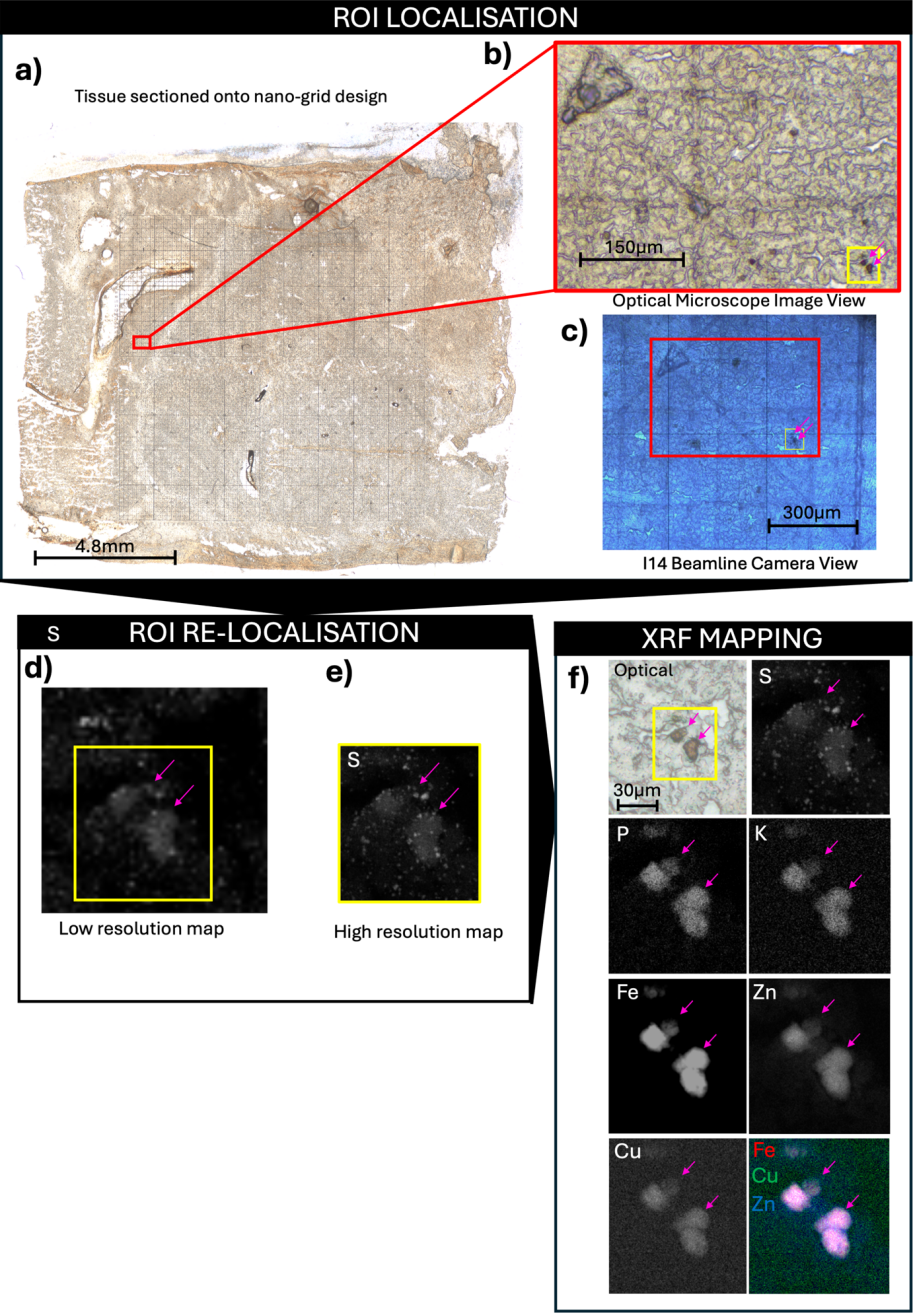
Correlative SXRF–optical workflow and ROI relocalization in a prestained Alzheimer’s disease brain section using the quartz finder grid system. Sections were mounted and stained on the quartz finder grid system. (a) Pathscanner optical overview of an Alzheimer’s disease brain section prestained with a modified hyperphosphorylated tau protocol prior to SXRF, mounted on the etched quartz grid. (b) Optical microscope image of the boxed area in (a). (c) I14 beamline camera image of the tissue area boxed in (a). The grid enables rapid navigation and precise recording of the selected region of interest (ROI, yellow box). (d, e) Staged SXRF mapping of ROI, using sulfur signal as an example. (d) Low-resolution confirmation scan (50 × 50 μm^2^, 1000 nm step size, and 0.1 s dwell) acquired to verify beam positioning, followed by (e) high-quality mapping of the refined ROI in (31 × 35 μm^2^, 200 nm step size, and 0.1 s dwell). (f) High-resolution optical image of hyperphosphorylated tau ROI, with corresponding SXRF elemental maps (S, P, K, Fe, Cu, and Zn) and a composite RGB image (Fe, green; Cu, green; and Zn, blue).

### 3.3 SXRF Alignment

In the nanoprobe format, the etched quartz grid was implemented primarily to aid ROI relocalization while maintaining compatibility with SXRF, as it provides a stable coordinate reference without introducing metal-derived background signal. In microprobe SXRF experiments, particularly for unstained tissue sections where morphological landmarks are limited, the grid supported both ROI relocalization and subsequent image realignment. Importantly, the grid pattern remained visible in SXRF elemental maps, facilitating registration between optical and SXRF data sets while contributing negligibly to measured elemental counts (Figure 6). This visibility persisted following thorough cleaning of the substrates prior to tissue mounting. We hypothesize that the apparent contrast arises from changes in X-ray scatter associated with the angled geometry of the laser-etched grooves, rather than from fluorescence originating from the grid itself.

**Figure 6:**
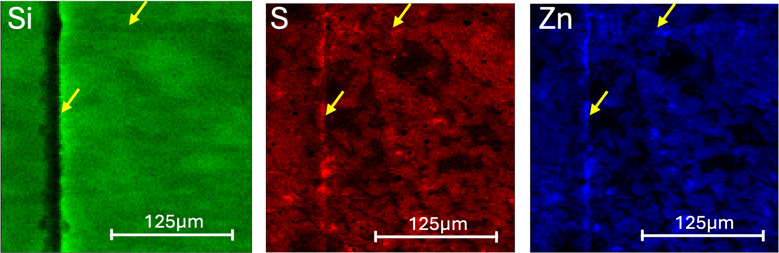
SXRF elemental maps of Si, S, and Zn demonstrating visibility of the etched quartz grid (vertical and horizontal grid lines, marked with yellow arrows) within the XRF data set, enabling ROI relocalization and image registration without contributing measurable elemental signal. Map area: 250 × 250 μm^2^; step size: 2 μm; and dwell time: 0.5 s per pixel (acquired at I18, Diamond Light Source).

## 4 Discussion

### 4.1 Spectroscopically Clean Fiduciary System for Metallomics Analysis

By employing a spectroscopically clean quartz substrate and an ablation-based etching process, we created a fiducial system with subppm levels of the metal elements that typically dominate conventional grids and other fiduciary systems. SXRF analysis rapidly confirmed the low and subtractable background signal in the context of a mapping experiment under helium conditions at a typical SXRF beamline (Figures 2and 3), enabling researchers to investigate biological concentrations of elements like copper and nickel without interference from the fiducial system itself.

### 4.2 Hierarchical Navigation across Micro- and Nano-SXRF Length Scales

In practice, nanoprobe compatibility is primary determined by beamline mounting constraints, so the nanoformat grid is broadly compatible with microprobe beamlines, whereas standard slide formats are not typically mountable at nanoprobes. The hierarchical architecture of this quartz grid system provides excellent navigational precision that directly addresses a critical challenge in spatial metallomics: the capability for nondestructive correlative analysis at subcellular resolutions in large tissue sections, entire organ slices, and model systems. The alternatives, where one is reliant on the introduction of signal-emitting markers or on the use of natural tissue landmarks such as vascular patterns or pigmented features, can be counterproductive, inaccurate, or even nonviable in tissue sections that lack clear features in the native state. This system directly solves the problem. The microgrid design (Figure 1b), produced on standard 25 × 75 mm quartz slides, provides a permanent, unambiguous coordinate system that can span an entire sample and can be adapted as required. This enables a practical and rigorous workflow where a researcher can seamlessly navigate from a macroscopic view of an entire tissue section on a standard light microscopy slide down to a specified 250 μm square using alphanumeric coordinates (e.g., “C5”). This integrated approach transforms large-area correlation from landscape-dependent, subjective interpretation into a coordinate-based system, eliminating the challenges of working across multiple instruments and spatial resolutions.

### 4.3 Multimodal Correlation on a Single Platform

The core innovation of this quartz grid system is that it provides the capability for unambiguous multimodal correlation on a single platform, as illustrated for an example of human brain tissue in Figures 4 and 5. The grid enables direct correlation of tissue morphology with multiple elemental distributions from SXRF imaging of native, unstained tissue, preserving the integrity of redox-sensitive and potentially labile metal pools. A particularly powerful application of this system is its ability to resolve the long-standing limitations associated with dependency on adjacent section analysis. The quartz substrate was able to withstand histological processing, enabling same-section correlation. Here, performing SXRF imaging and correlative histology on precisely the same tissue region enables extended interpretation across length scales from cellular to multilayer and by anatomical region. The permanent fiducial grid provides a reproducible coordinate frame for rapid ROI relocalization across instruments and quantitative overlay using control points (grid intersections/markers), reducing user-dependent variability compared with relying solely on tissue edges or native irregularities, particularly in feature-poor tissue. This nondestructive multimodal capability is particularly important for the investigation of isolated features or specific cell populations where precise spatial correlation is essential to biological interpretation.

### 4.4 Scope for Utilization at Beamlines Internationally

The example designs illustrated in this Technical Note are straightforward for utilization on beamlines where imaging does not depend on transmission of wavelengths attenuated by quartz and that have sample holders that accommodate standard-sized light microscopy slides. However, the same concept, employing smaller-format quartz substrates with modified designs, could be used as required. The applicability of our proposed design will depend on the specific design of the sample holders at each facility. Selected examples of current beamlines where the system might be applied include the ESRF ID21 beamline, which offers scanning X-ray microscopy with reported spatial resolution of 1 μm and a motorized stage capable of ± 10 mm motion in the *X–Y* plane, enabling millimeter-scale scan ranges.^19^ ESRF ID21 is also equipped with an on-axis microscope camera for precise sample alignment. Additionally, the Australian Synchrotron XFM beamline supports elemental mapping of thin sections mounted on slides.^18^ The technical examples reported here were performed at Diamond Light Source but could be undertaken at other facilities in order to combine micro- and nanoscale XRF imaging, or to employ these modalities separately in reflective fluorescence geometries with supporting optical visualization. The approach demonstrated is therefore not a facility-specific solution, but a transferable fiducial and coordinate framework.

## 5 Conclusions

The development of this hierarchically patterned fiducial system aims to address uncertainties in correlative metallomics studies, addressing present challenges in spatial alignment and data interpretation. The etched grids on quartz substrates enable same-section correlation with a clear coordinate system, without the introduction of confounding XRF signal. This ensures that elemental distributions can be correctly assigned to specific cellular and subcellular structures within large-area sections, rather than having to rely on inference from adjacent stained sections. By providing a scalable fiducial system from standard slide to coverslip formats, we enable precise correlation for large tissue samples, integrating macroscopic tissue context with cellular-level distribution and subcellular localization.

The correlative workflow presented in this Technical Note, connecting optical microscopy, SXRF, and histology, represents one application for this system. Although the current requirement for a femtosecond laser etching process presents a cost and accessibility barrier, the etched grid system itself is inherently adaptable. For example, when applied to other spectroscopically clean substrates with appropriate material properties and thicknesses that can also tolerate the energy deposition required for laser etching, the grid system could conceivably be extended to support techniques such as Raman spectromicroscopy, mass spectrometry imaging, and super-resolution fluorescence microscopy. This offers a versatile tool for the next generation of correlative imaging, with applicability to diverse topics such as metallomics, toxicology, and biomedical implant development in the chemical and biomedical imaging field.

## Data Availability

The data that support the findings in this study are available in the Warwick Research Archive Portal (WRAP) repository at [https://wrap.warwick.ac.uk/id/eprint/197379/]
